# Salvage of Infected Prosthetic Grafts at the Groin or Thigh Using Muscle Flap Coverage

**DOI:** 10.3400/avd.oa.20-00123

**Published:** 2020-12-25

**Authors:** Kiyoshi Tanaka, Shinsuke Mii, Masaru Ishida, Atsushi Guntani, Eisuke Kawakubo, Shinichi Tanaka, Ryosuke Yoshiga, Jin Okazaki

**Affiliations:** 1 Department of Vascular Surgery, Kokura Memorial Hospital, Kitakyushu, Fukuoka, Japan; 2 Department of Vascular Surgery, Saiseikai Yahata General Hospital, Kitakyushu, Fukuoka, Japan; 3 Department of Vascular Surgery, Steel Memorial Yawata Hospital, Kitakyushu, Fukuoka, Japan

**Keywords:** graft infection, prosthetic graft, muscle flap, sartorius muscle, gracilis muscle

## Abstract

**Objectives:** This study aimed to evaluate early- and long-term outcomes in patients who undergo muscle flap coverage (MFC) for prosthetic graft infections (PGIs) at the groin or thigh.

**Materials and Methods:** We retrospectively retrieved and analyzed data on infected wound cures, recurrence, graft and limb salvage, and survival of patients who underwent MFC for PGI at the groin or thigh between 2000 and 2018.

**Results:** There were eight patients in our cohort: six had groin PGIs and two had thigh PGIs. Moreover, of these patients, seven were treated from sartorius muscles and one from a gracilis muscle. The indicated wounds healed in all eight patients, but two patients died during hospitalization. Three patients suffered recurrence within 8 months, one of which overcame the infection and achieved wound cure without graft removal, with negative pressure wound therapy. No patients lost their limbs during the follow-up term (mean, 24 months; range, 1–60 months). Finally, four patients (50%) survived without removal of the infected graft for longer than 2 years.

**Conclusion:** MFC can be a curative treatment for PGI, but there remains a possibility of a recurring infection thereafter.

## Introduction

Infection is one of the most serious complications of prosthetic grafts. It can have catastrophic results without adequate treatment. The groin is the most common site for postoperative graft infection in vascular surgery. Usually, optimal treatment involves removing the infected graft and extra-anatomical bypass to detour the infected region, with the use of appropriate antibiotics. Muscle flap coverage (MFC) is an alternative procedure to maintain blood flow without graft removal; its effectiveness has been demonstrated in some reviews.^1–3)^ We have performed MFC for prosthetic graft infections (PGIs) at the groin or thigh in eight patients. The clinical features of these patients are summarized in this study, and their early- and long-term outcomes are discussed with respect to PGI treatment issues.

## Materials and Methods

The Institutional Review Board of Kokura Memorial Hospital, Kitakyushu City, Japan, approved the study design (No. 20072801; July 31, 2020).

### Patients

Between April 2000 and June 2018, eight patients (seven men and one woman) underwent MFC for PGI (six at the groin and two at the thigh) at Steel Memorial Yawata Hospital, Kokura Memorial Hospital, and Saiseikai Yahata General Hospital. We retrospectively reviewed their data, such as preoperative characteristics, surgical information, and outcomes.

We obtained approval documents for this paper from the two living patients. Since the other six patients are known to have died, or have been missing for more than 5 years, their written approval was judged to be unnecessary by the Institutional Review Board of Saiseikai Yahata General Hospital, Kitakyushu City, Japan.

### Strategy for graft infection

Graft sites that produced purulent discharges were irrigated twice a day with warmed saline containing 4% povidone-iodine and were treated with appropriate intravenous antibiotics. These procedures were continued, together with debridement of any necrotic tissue, until the infection localized, as determined by clinical findings and CT scan. When the purulent discharge from the skin fistula continued for more than 1 week, FC could then be planned. However, graft salvage was not considered suitable for patients who suffered graft occlusion, infection spreading throughout the length of the graft, bleeding (which implies anastomotic line infection), or systemic sepsis.

### Surgical procedures and postsurgical management

**Figure 1:**
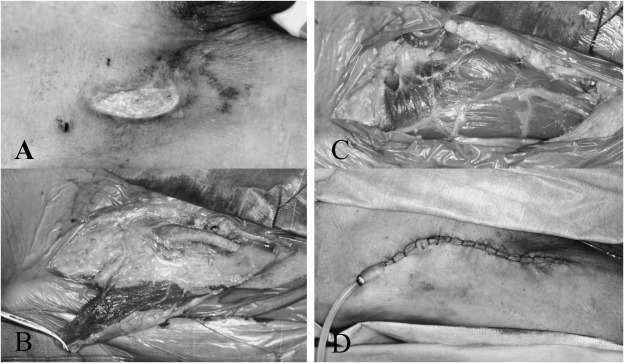
Fig. 1 Sartorius muscle flap coverage. (**A**) Before the operation. (**B**) Debridement of infected subcutaneous tissue around femoropopliteal bypass graft, and sartorius muscle severed at the origin. (**C**) Graft covered with the twisted sartorius muscle. (**D**) Wound closure with insertion of closed suction drain.

For the sartorius muscle flap (**Fig. 1**), under general anesthesia, a skin incision that included the infectious skin was made from the iliac spine to the upper thigh. The subcutaneous tissues around the graft were completely excised to the healthy area. After irrigation with warm saline that included aminoglycoside antibiotics, and complete hemostasis, the proximal part of the sartorius muscle was isolated and its origin severed at the iliac spine. The sartorius muscle was twisted medially and sutured to the tissues surrounding the prosthetic graft to contact intimately with the graft surface. The wound was then closed after inserting a closed suction drain, placed on the muscle flap.

**Figure 2:**
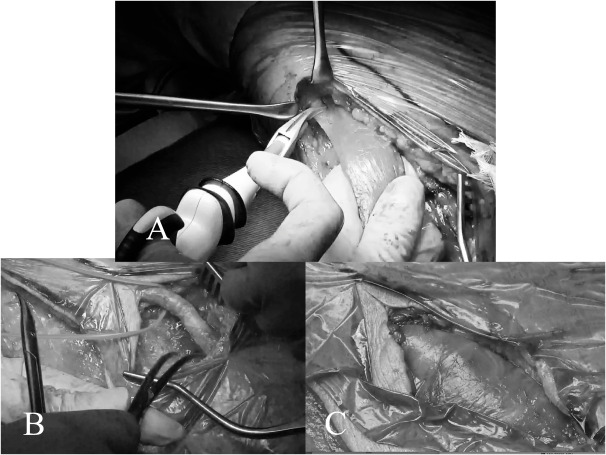
Fig. 2 Gracilis muscle flap coverage. (**A**) Division of the gracilis muscle. (**B**) Debridement of infected subcutaneous tissue around femoroperoneal bypass graft with Miller cuff at the upper thigh. (**C**) Graft covered with the retroflexed gracilis muscle.

For the gracilis muscle flap (**Fig. 2**), a skin incision was made at the medial thigh over the gracilis muscle. The subcutaneous tissue and deep fascia were dissected to the gracilis muscle, which was mobilized to the tendinous portion at the knee and divided there. The distal portion of the mobilized muscle was retroflexed toward the infected graft. The procedures thereafter were the same as for the sartorius muscle above.

After surgery, appropriate antibiotics (i.e., sensitive to the detected organism) were intravenously administered for at least 1 week and, when necessary, continued until the laboratory data and physical findings indicated that the infection had resolved. Subsequently, oral antibiotics were prescribed for as long as the attending doctor considered necessary. The closed suction drain was removed when the discharge volume decreased to 30 mL per day.

### Follow-up observation after discharge

Patients were followed-up for 1–3 months to check their surgical wounds, including graft patency, using physical examinations, duplex ultrasound, and laboratory data. If infection recurred, the healthcare provider recommended that the patient be hospitalized.

### Endpoints

Graft salvage and death were set as primary endpoints, while cure of the indicated wound, infection recurrence, and additional treatment after infection recurrence were set as secondary ones. A final review was conducted in June 2020, and outcomes were evaluated up to 2 years following MFC.

## Results

The eight patients’ clinicopathological details and re-, intra-, and postsurgical information are summarized in **Tables 1** and **2**, and the postsurgical outcomes details are presented in **Table 3**. All patients (100%) achieved healing of their indicated wounds. Two patients (25%, Patients 1 and 2) died with viable grafts during hospitalization for the indicated MFC. Both showed moderate to severe hypoalbuminemia and could not walk unassisted before their MFCs. They seemed unable to tolerate removal of the infected grafts and the subsequent revascularization, apparently due to their frailty. Three of the six patients who were eventually discharged (Patients 3, 4, and 8) had infection recurrences 74, 243, and 122 days, respectively, following their MFCs. Two patients had their grafts removed, and one of which (Patient 3) underwent simultaneous obturator bypass. Patient 4 underwent graft removal, only because he was nourished through a nasogastric tube due to a subsequent stroke. Another patient (Patient 8) overcame a recurrent infection with negative pressure wound therapy (NPWT) without graft removal. Finally, four patients (50%), including Patient 8, were alive without graft removal 2 years after MFC.

**Table 1:** Table 1 Summary of clinicopathological characteristics in eight patients who underwent muscle flap coverage for infected prosthetic grafts

Case	Age Sex	Initial op.	Basic disease	Site of infection	Material of graft	Term from initial op. to Dx of infection	Term from Dx of infection to MFC	Bacteria	Comorbidity
In-hospital death									
1	67 male	FP bypass	ASO (R-5)	Groin	Dacron	16 days	15 days	MRSA	DM, CKD
2	81 male	FF bypass of EVAR	AAA	Groin	ePTFE	26 days	29 days	Entero	IHD, COPD
								MRSA	
Graft loss due to recurrence of infection									
3	72 male	FF bypass	ASO (R-4)	Groin	Dacron	40 months	20 days	None	CKD
4	83 male	FF bypass	ASO (R-4)	Groin	Dacron	28 days	48 days	MRSE	DM, HD, CVD
Life survival with graft salvage for longer than 2 years									
5	67 male	FF bypass	ASO (R-4)	Groin	ePTFE	20 months	19 days	MSSA	Psoas abscess
6	72 male	Ana. aneu. of Ax-F bypass	ASO (R-1)	Groin	Dacron	14 days	20 days	MRSA	CVD, malignancy
7	89 male	Thrombectomy of F-PE bypass	ALI	Thigh	ePTFE	16 days	11 days	MRSA	None
8	74 female	AKFP bypass	ASO (R-5)	Thigh	ePTFE	3 months	16 days	MRSA	COPD, steroid

AAA: abdominal aortic aneurysm; AKFP: above knee femoro-popliteal; ALI: acute limb ischemia; Ana. aneu.: anastomotic aneurysm; ASO: arteriosclerosis obliterans; Ax-F: axillofemoral; CKD: chronic kidney disease; COPD: chronic obstructive pulmonary disease; CVD: cerebrovascular disease; DM: diabetes mellitus; Dx: diagnosis of infection; Entero.: enterococcus; ePTFE: expanded polytetrafluoroethylene; EVAR: endovascular aneurysm repair; FF: femorofemoral crossover; FP: femoropopliteal; F-PE: femoroperoneal; HD: hemodialysis; IHD: ischemic heart disease; MFC: muscle flap coverage; MRSA: methicillin-resistant *Staphylococcus aureus*; MRSE: methicillin-resistant *Staphylococcus epidermidis*; MSSA: methicillin-sensitive *Staphylococcus aureus*; op.: operation; R: Rutherford classification

**Table 2:** Table 2 Summary of pre-, intra-, and postsurgical information for eight patients

Case	CRP	WBC (/μL)	Lymphocyte (%)	Alb (g/dL)	Used muscle	Op. time	Blood loss	Intravenous antibiotics	Oral antibiotics	Total duration of antibiotics
In-hospital death										
1	1.53	9500	17.6%	2.3	Sartorius	45 min	Little	VCM	—	3 weeks
2	10.7	8000	30%	2.5	Sartorius	67 min	Little	MINO, DAP, TAZ/PIPC	—	4 weeks
Graft loss due to recurrence of infection										
3	1.41	5000	28.8%	3.5	Sartorius	70 min	40 g	FMOX	LVFX	4 weeks
4	6.9	7200	17.0%	2.6	Sartorius	45 min	Little	VCM, ABK	RFP	16 weeks
Life survival with graft salvage for longer than 1 year										
5	NR	6600	NR	3.4	Sartorius	115 min	100 g	ABPC/SBC, CLDM	No	8 weeks
6	1.86	4700	29.6%	3.2	Sartorius	50 min	50 g	DAP, MINO	MINO or LVFX	4 weeks
7	6.18	8500	18.0%	3.0	Gracilis	103 min	150 g	DAP or VCM or AMK	No	6 weeks
8	0.46	5300	17.7%	3.4	Sartorius	49 min	30 g	DAP	No	4 weeks

ABPC/SBC: ampicillin/sulbactam; Alb: serum albumin; ABK: arbekacin; AMK: amikacin; CLDM: clindamycin; CRP: C-reactive protein; DAP: daptomycin; FMOX: flomoxef; LVFX: levofloxacin; MINO: minocyclin; NR: no record; RFP: rifampicin; TAZ/PIPC: tazobactam/piperacillin; VCM: vancomycin; WBC: white blood cells

**Table 3:** Table 3 Postsurgical outcomes in eight patients who underwent muscle flap coverage for infected prosthetic grafts

Case	Hospitalization days after MF	Months from MF to recurrence date	Months from MF to graft loss date	Additive procedure for limb salvage	Life	Death cause	Months from MF to final prognosis
In-hospital death							
1	58 days	No	No	—	Dead	Sepsis due to foot infection	58 days
2	31 days	No	No	—	Dead	ARDS	31 days
Graft loss due to recurrence of infection							
3	22 days	74 days	94 days	Obtulator bypass	Dead	Cerebral hemorrhage	16 months
4	24 days	243 days	265 days	Graft removal with no reconstruction	Dead	MNMS	9 months
Life survival with graft salvage for longer than 1 year							
5	30 days	No	No	—	Alive		43 months
6	19 days	No	No	—	Dead	Pneumonia	60 months
7	35 days	No	No	—	Alive		24 months
8	31 days	122 days	No	NPWT	Alive		37 months

ARDS: acute respiratory distress syndrome; MF: muscle flap; MNMS: myonephropathic metabolic syndrome; NPWT: negative pressure wound therapy

The group of patients with the better prognosis, who were alive with graft salvage at 2 years after MFC (Patients 5, 6, 7, and 8), and those with the worse prognosis, who died within 1 year (Patients 1, 2, 3, and 4), showed no marked differences in clinicopathological characteristics, surgical findings, or duration of antibiotic use. However, three of four patients in the worse prognosis group had serum albumin concentrations <3.0 g/dL, had chronic kidney disease (CKD) requiring hemodialysis, and were non-ambulatory, whereas all patients in the better prognosis group had albumin concentrations ≥3.0 g/dL, had no CKD, and were ambulatory.

## Discussion

The goals of MFC for PGI in the groin are adequate coverage of the underlying prosthetic graft material and elimination or control of infection in a single procedure without discontinuing blood flow to the lower extremity.^4)^ Several muscles can be used to reach these goals,^2)^ with the sartorius muscle flap most frequently reported, with satisfactory outcomes.^5–9)^ A theoretical advantage of using this muscle is that no second soft tissue defect is required to harvest the donor flap.^1)^ Although Ali et al. found that this muscle was often ischemic or inflamed from invasive infection with an occluded superficial femoral artery (SFA),^10)^ Töpel et al. found no significant difference in muscle viability, infection recurrence, loss of vascular reconstruction, or limb salvage between patent and occluded SFAs.^6)^ In addition to the sartorius muscle, use of the rectus femoris muscle^1,2,9,11,12)^ and the gracilis muscle^4,10,13)^ has been widely reported. These muscles are located away from the active infection at the infected graft.^4)^ In addition, ischemia is not a concern if the SFA is occluded because the vessel is nourished via a branch from the deep femoral artery.^2,10)^ The rectus femoris muscle is bulky and long and has a wide arc of rotation; it is a very mobile flap for use as a graft overlap.^2)^ Wübbeke et al. described in a systemic review that the superiority of sartorius and rectus femoris muscles with respect to amputation or mortality was not demonstrated; however, the vascular graft loss rate was lower after rectus femoris muscle flap reconstruction.^1)^ Mirzabeigi et al. found no significant difference in rates for complications or graft salvage and concluded that the sartorius muscle was an effective first-line approach, as rectus femoris muscle flaps might increase functional morbidity and necessitate second donor sites.^5)^ Morasch et al. also found that using the rectus femoris muscle caused more morbidity and functional deficits than using the gracilis muscle.^4)^

MFCs from these muscles initially had a high success rate in the indicated wound healing and high limb salvage rates.^4,6–12)^ However, early mortality rates ranged from 0% to 24%,^4,6,8,10–12)^ which suggests that these procedures are too invasive for fragile patients with graft infections; the indications should be carefully determined. Since patients who suffer from graft infection are often nutritionally depleted, with significant comorbidities, graft infection is not their only issue, and the indications for this invasive treatment for graft infection should be determined carefully.

NPWT is another method for graft salvage. Berger et al. and Dosluoglu et al., respectively, showed that among patients with Szilagyi III wounds, 14 of 17 patients (82%) and 10 of 12 patients (83%) achieved wound healing.^14,15)^ Conversely, May et al. reported that six of seven patients with graft losses underwent NPWT before MFC and concluded that MFC performed soon after initial wound complication presentation improved vascular graft salvage.^16)^ Svensson et al. reported 2 early bleeding and 3 late pseudoaneurysms in 33 patients with vascular groin infections who were treated by NPWT and concluded that vascular graft infections of the groin treated with NPWT were at greater risk of developing infection-related complications, which were associated with higher rates of amputation and death.^17)^

We did not always attempt graft salvage by MFC in patients with PGIs. If a patient was able to tolerate the invasive surgery, the infected graft was excised, and subsequent revascularization^18–20)^ was added whenever possible. This most desirable treatment was also performed if graft salvage by MFC or NPWT failed or if graft infection recurred.

In this series, four patients were alive with graft salvage 2 years after MFC, and the other four died within 1 year. Obvious differences between the better and the worse prognosis group were serum albumin concentrations on the day before surgery, CKD, and the ability to walk unaided. These variables are reportedly independent predictors for ischemic wound healing or life-threatening outcomes in patients who undergo open bypass for critical limb ischemia.^21,22)^ Therefore, these factors might be helpful in deciding on MFC.

As observed in this series, recurrence of graft infection or graft occlusion will occur even if the MFC operative wound appears to achieve healing with a patent graft. Accordingly, we strongly emphasize the importance of lifelong surveillance.

## Conclusion

MFC is a useful procedure for PGI at the groin or thigh. However, recurrence might occur at any time, even when the indicated wound has healed.
